# Correction: Small Intestine Bacterial Overgrowth is associated with increased Campylobacter and epithelial injury in duodenal biopsies of Bangladeshi children

**DOI:** 10.1371/journal.pntd.0013354

**Published:** 2025-07-23

**Authors:** The PLOS Neglected Tropical Diseases Staff

## Notice of republication

This article was republished on July 11, 2025, to correct errors in the fourth author’s name that were introduced during the typesetting process. The correct name is: Ning-Jiun Jan. The publisher apologizes for the errors. Please download this article again to view the correct version. The originally published, uncorrected article and the republished, corrected articles are provided here for reference.

## Supporting information

S1 File Originally published, uncorrected article. (PDF)

S2 File Republished, corrected article. (PDF)
