# Supplementary figures and images for: Small Intestine Bacterial Overgrowth is associated with increased Campylobacter and epithelial injury in duodenal biopsies of Bangladeshi children
Source: PLoS Negl Trop Dis. 2024 Mar 27;18(3):e0012023. doi: 10.1371/journal.pntd.0012023 (PMC11020352; doi:10.1371/journal.pntd.0012023)

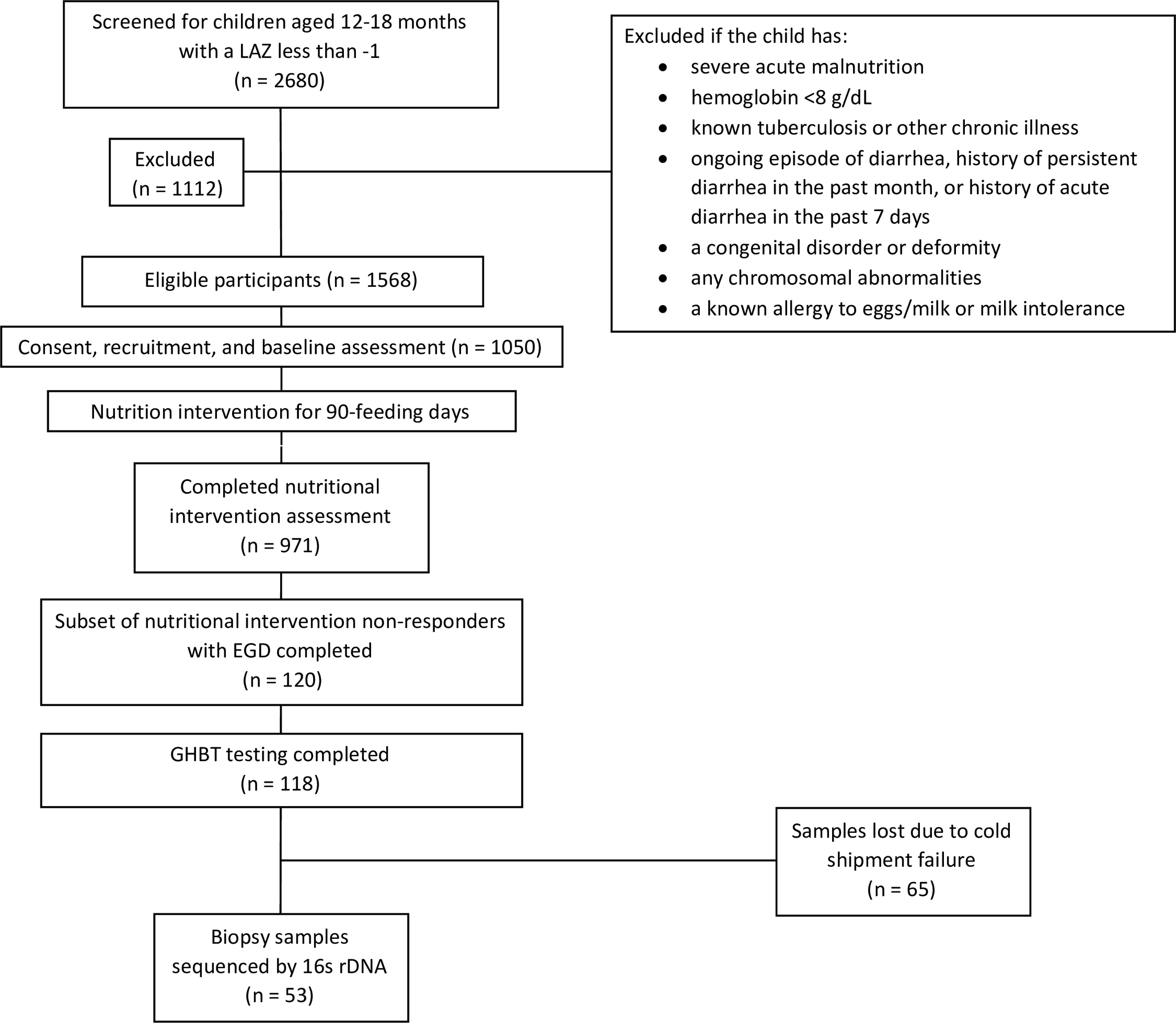

Supplement: S1 Fig — 118 children enrolled in the original Bangladesh Environmental Enteric Diseases (BEED) study had glucose-hydrogen breath testing for SIBO with 53 of those having 16s rRNA sequencing conducted on duodenal biopsy samples. (TIF) [file pntd.0012023.s001.tif]

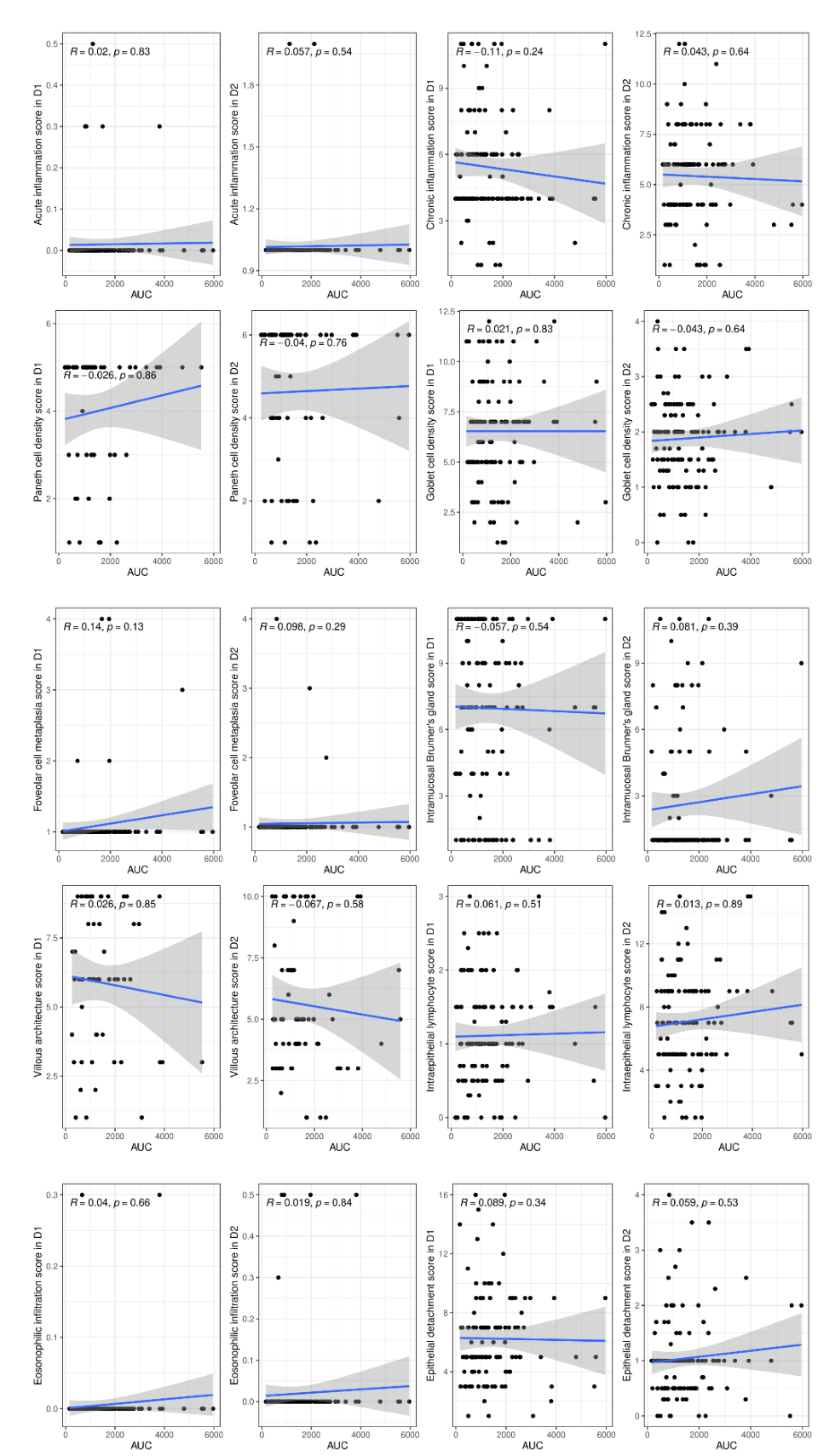

Supplement: S2 Fig — Enterocyte injury (Fig 1) was the only significant feature. No other features demonstrated a significant association. (TIF) [file pntd.0012023.s002.tif]
