# Supplementary material for: Small Intestine Bacterial Overgrowth is associated with increased Campylobacter and epithelial injury in duodenal biopsies of Bangladeshi children
Source: PLoS Negl Trop Dis. 2024 Mar 27;18(3):e0012023. doi: 10.1371/journal.pntd.0012023 (PMC11020352; doi:10.1371/journal.pntd.0012023)
Supplement: S1 Table — (DOCX) [file pntd.0012023.s003.docx]

**Supplementary Table 1:**

| **Acute (neutrophilic) inflammation** | **0**: PMNs may be present in vessels or in lamina propria, but there is no intraepithelial infiltration (cryptitis,  villitis) |
| --- | --- |
|  | **1**: 1–2 foci of epithelial neutrophilic infiltration or crypt microabscesses |
|  | **2**: > 2 foci of epithelial neutrophilic infiltration or crypt microabscesses, but 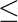 50% of mucosa involved |
|  | **3**: > 50% of mucosa involved by epithelial neutrophilic infiltration |
| **Eosinophil infiltration** | **0:** No increase in eosinophils (highly scattered in lamina propria, no intravillous or intercryptal space with > 5 eosinophils) |
|  | **1:** Increased eosinophils (intravillous or intercryptal space with > 5 eosinophils) involving 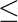 50% of mucosa, with no eosinophilic crypt microabcesses |
|  | **2:** Increased eosinophils (intravillous or intercryptal space with > 5 eosinophils) involving > 50% of mucosa, or up to 1 focus of eosinophilic epithelial infiltration or crypt microabcesses per mucosal fragment |
|  | **3:** > 2 foci of eosinophilic epithelial infiltration or crypt microabcesses in any mucosal fragment |
| **Chronic inflammation** | **0:** No qualitative increase in mononuclear inflammatory cells (MIC) in lamina propria. Majority of villous bases contain < 3 MIC across, on average. |
|  | **1:** Increased MIC, based on villous base displaying 3–5 MIC across, on average. |
|  | **2:** Increased MIC, based on villous base displaying 6–10 MIC across, on average. |
|  | **3:** Increased MIC, based on villous base displaying > 10 lymphocytes on average. |
| **Intraepithelial lymphocytes** | **0:** Lymphocyte/epithelial ratio < = 20% in any area |
|  | **1:** Lymphocyte/epithelial ratio > 20%, but 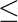 50%, in < = 50% of mucosa |
|  | **2:** Lymphocyte/epithelial ratio > 20%, but 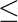 50%, in > 50% of mucosa |
|  | **3:** Lymphocyte/epithelial ratio > 50% in 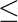 50% of mucosa |
|  | **4:** Lymphocyte/epithelial ratio > 50% in > 50% of mucosa |
| **Villous architecture** | **0:** Majority of villi are > 3 crypt lengths long |
|  | **1:** Villi are 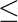 3 but > 1 crypt length long, with abnormality in 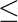 50% of mucosa. |
|  | **2:** Villi are 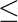 3 but > 1 crypt length long, with abnormality in > 50% of mucosa |
|  | **3:** Villi absent, or 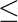 1 crypt length long, with abnormality in 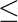 50% of mucosa |
|  | **4:** Villi absent, or 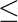 1 crypt length long, with abnormality in > 50% of mucosa |
| **Intramucosal Brunner glands** | **0:** Brunner glands are in submucosa, but are not observed above the muscularis mucosae |
|  | **1:** 1–2 foci, none involving > 5 crypt bases |
|  | **2:** 3–5 foci, none involving > 5 crypt bases |
|  | **3:** > 5 foci, or any area of intramucosal Brunner glands involving > 5 crypt bases |
| **Foveolar cell metaplasia** | **0:** Only absorptive enterocytes and goblet cells observed on villi, no evidence of foveolar cells |
|  | **1**: Foveolar mucin cells observed, usually on the tips of the villi; 1–2 villous tips involved |
|  | **2:** Foveolar mucin cells observed, usually on the tips of the villi; 3–5 villous tips involved |
|  | **3:** Foveolar mucin cells observed, usually on the tips of the villi; > 5 villous tips involved |
| **Goblet cell density** | **0:** Normal goblet cell density (at least 1 goblet cell per 20 enterocytes) in all evaluable mucosal epithelial layer |
|  | **1:** Decreased goblet cells (< 1/20 enterocytes) in 1–25% of evaluable mucosal epithelium |
|  | **2:** Decreased goblet cells (< 1/20 enterocytes) in 26–50% of evaluable mucosal epithelium |
|  | **3:** Decreased goblet cells (< 1/20 enterocytes) in 51–75% of evaluable mucosal epithelium |
|  | **4:** Decreased goblet cells (< 1/20 enterocytes) in 76–100% of evaluable mucosal epithelium |
| **Paneth cell density** | **0:** 2: 5 Paneth cells/ crypt, on average |
|  | **1:** 2–4 Paneth cells/ crypt, on average |
|  | **2:** < 2 Paneth cell/crypt, involving 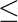 50% of crypt bases |
|  | **3:** < 2 Paneth cell/crypt, involving > 50% of crypt bases |
| **Enterocyte injury** | **0:** Majority of enterocytes (90%) show tall columnar morphology |
|  | **1:** Enterocytes show low columnar (< 2:1 L:W ratio), cuboidal or flat morphology, in 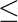 50% of mucosa |
|  | **2:** Enterocytes show low columnar (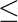2:1 L:W ratio), cuboidal or flat morphology, in > 50% of mucosa |
|  | **3:** Any area of mucosal erosion/ulceration |
| **Epithelial detachment** | **0**: Complete coverage of mucosal surface by epithelial cells |
|  | **1**: Surface epithelium missing or detached from < 25% of mucosa |
|  | **2**: Surface epithelium missing or detached from 25–50% of mucosa |
|  | **3**: Surface epithelium missing or detached from 51–75% of mucosa |
|  | **4**: Surface epithelium missing or detached from > 75% of mucosa |
| Adapted from: Liu, Ta-Chiang, et al. "A novel histological index for evaluation of environmental enteric dysfunction identifies geographic-specific features of enteropathy among children with suboptimal growth." PLoS neglected tropical diseases 14.1 (2020): e0007975. (22) | |
