# Supplementary material for: Small Intestine Bacterial Overgrowth is associated with increased Campylobacter and epithelial injury in duodenal biopsies of Bangladeshi children
Source: PLoS Negl Trop Dis. 2024 Mar 27;18(3):e0012023. doi: 10.1371/journal.pntd.0012023 (PMC11020352; doi:10.1371/journal.pntd.0012023)
Supplement: S2 Table — (DOCX) [file pntd.0012023.s004.docx]

**Supplementary Table 2:** The primers used for V4 amplification, first published by Kozich et al. (31).

| +  strand | ………….CTTCCACTTAAATGAGACTT GTGCCAGCMGCCGCGGTAA……………..ATTAGAWACCCBDGTAGTCC ATACAGGTGAGCACCTTGTA… |
| --- | --- |
| -strand | ………….GAAGGTGAATTTACTCTGAA CACGGTCGKCGGCGCCATT……………..TAATCTWTGGGVHCATCAGG TATGTCCACTCGTGGAACAT… |
