# Supplementary material for: Correction: Small Intestine Bacterial Overgrowth is associated with increased Campylobacter and epithelial injury in duodenal biopsies of Bangladeshi children
Source: PLoS Negl Trop Dis. 2025 Jul 23;19(7):e0013354. doi: 10.1371/journal.pntd.0013354 (PMC12286358; doi:10.1371/journal.pntd.0013354)
Supplement: S2 File — (PDF) [file pntd.0013354.s002.pdf]

RESEARCH ARTICLE

Small Intestine Bacterial Overgrowth is associated with increased *Campylobacter* and epithelial injury in duodenal biopsies of Bangladeshi children

Shah Mohammad Fahim[1,2‡], Jeffrey R. Donowitz[3,4,5‡*], Ekaterina Smirnova[6], Ning-Jiun Jan[4], Subhasish Das[1], Mustafa Mahfuz[1], S. M. Abdul Gaffar[1], William A. PetriJr[4], Chelsea Marie[4‡], Tahmeed Ahmed[1,7,8,9‡]

1 Nutrition and Clinical Services Division, International Centre for Diarrhoeal Disease Research, Bangladesh (icddr,b), Dhaka, Bangladesh, **2** Division of Nutritional Sciences, Cornell University, Ithaca, New York, United States of America, **3** Division of Pediatric Infectious Diseases, Children's Hospital of Richmond at Virginia Commonwealth University, Richmond, Virginia, United States of America, **4** Division of Infectious Diseases and International Health, University of Virginia, Charlottesville, Virginia, United States of America, **5** Division of Pediatric Infectious Diseases, University of Virginia, Charlottesville, Virginia, United States of America, **6** Department of Biostatistics, Virginia Commonwealth University, Richmond, Virginia, United States of America, **7** Office of the Executive Director, International Centre for Diarrhoeal Disease Research, Bangladesh (icddr,b), Dhaka, Bangladesh, **8** Department of Global Health, University of Washington, Seattle, Washington, United States of America, **9** Department of Public Health Nutrition, James P Grant School of Public Health, BRAC University, Dhaka, Bangladesh

‡ SMF and JRD share first authorship on this work. CM and TA are joint senior authors on this work.
* jrd9d@virginia.edu

OPEN ACCESS

Citation: Fahim SM, Donowitz JR, Smirnova E, Jan N-J, Das S, Mahfuz M, et al. (2024) Small Intestine Bacterial Overgrowth is associated with increased *Campylobacter* and epithelial injury in duodenal biopsies of Bangladeshi children. PLoS Negl Trop Dis 18(3): e0012023. https://doi.org/10.1371/journal.pntd.0012023

Editor: James A. Berkley, KEMRI-Wellcome Trust Research Programme, KENYA

Received: June 8, 2023

Accepted: February 26, 2024

Published: March 27, 2024

Copyright: © 2024 Fahim et al. This is an open access article distributed under the terms of the Creative Commons Attribution License, which permits unrestricted use, distribution, and reproduction in any medium, provided the original author and source are credited.

Data availability statement: All data, metadata, and code utilized in this study are available on Open Science Framework at https://osf.io/6mbp3/?view_only=1e0acd7a902644a58be15e4551926f81.

Funding: Funding was provided by Bill & Melinda Gates Foundation (https://www.gatesfoundation.org/) [OPP1136751] to TA;

Abstract

Small intestine bacterial overgrowth (SIBO) has been associated with enteric inflammation, linear growth stunting, and neurodevelopmental delays in children from low-income countries. Little is known about the histologic changes or epithelial adherent microbiota associated with SIBO. We sought to describe these relationships in a cohort of impoverished Bangladeshi children. Undernourished 12-18-month-old children underwent both glucose hydrogen breath testing for SIBO and duodenoscopy with biopsy. Biopsy samples were subject to both histological scoring and 16s rRNA sequencing. 118 children were enrolled with 16s sequencing data available on 53. Of 11 histological features, we found that SIBO was associated with one, enterocyte injury in the second part of the duodenum (R = 0.21, p = 0.02). SIBO was also associated with a significant increase in *Campylobacter* by 16s rRNA analysis (Log 2-fold change of 4.43; adjusted p = 1.9 x 10^{-6}). These findings support the growing body of literature showing an association between SIBO and enteric inflammation and enterocyte injury and further delineate the subgroup of children with environmental enteric dysfunction who have SIBO. Further, they show a novel association between SIBO and *Campylobacter*. Mechanistic work is needed to understand the relationship between SIBO, enterocyte injury, and *Campylobacter*.

The National Institutes of Health (https://www.nih.gov/) [NIH K23 HD097282]to JRD; and [NIH R01 AI043596] to WAP. The funders had no role in study design, data collection and analysis, decision to publish, or preparation of the manuscript. SMF, SD, MM, SMAD, WAP, CM, and TA received salary support from the Bill & Melinda Gates Foundation. JRD and WAP received salary support from the National Institutes of Health.

Competing interests: The authors have declared that no competing interests exist.

Author summary

Small intestine bacterial overgrowth (SIBO) is common in low- and middle-income country children with an incidence of up to 30% in some populations. SIBO has been documented as early as 18 weeks of age using the glucose-hydrogen breath test. SIBO is associated with future linear growth shortfalls and decreased language scores on neurodevelopmental testing. Other studies have linked SIBO to enteric inflammation but no work has directly examined histologic associations with the hydrogen breath test. Further, no work linking the duodenal adherent microbiota to breath testing has been published to date.

We conducted glucose-hydrogen breath testing and duodenoscopy with biopsy on Bangladeshi toddlers. Biopsy samples were subject to both histological scoring and 16s rRNA sequencing. SIBO was associated with enterocyte injury and with a significant increase in *Campylobacter* species in biopsy samples. These findings support the growing body of literature showing an association between SIBO and enteric inflammation and enterocyte injury. Further, they show a novel association between SIBO and *Campylobacter* species.

Introduction

Environmental enteric dysfunction (EED) is an asymptomatic syndrome of small bowel dysfunction with adverse impact on childhood growth and development [1,2]. It is classically characterized by the alteration of small intestinal architecture including villous atrophy, elongation of crypts, and increased infiltration of inflammatory cells in the lamina propria although recent work to more fully describe the histological changes in EED has also described Paneth and Goblet cell abnormalities in patients with EED [2,3]. EED involves enteric inflammation, loss of barrier integrity, and nutrient malabsorption [4,5]. It also involves a dysbiosis of the small intestine [6–9]. This syndrome is highly prevalent among the residents of low- and middle-income countries residing in unsanitary living conditions [10,11]. Poor water, sanitation, and hygiene practices along with the consumption of fecally contaminated food and water lead to sustained exposure to enteric pathogens [12]. This alters the local immune system and is associated with an intestinal dysbiosis [2,7,10].

Small intestine bacterial overgrowth (SIBO) is a particular dysbiosis which has been documented in a subset of children with EED [9,13,14]. Glucose-hydrogen-breath-testing (GHBT) is the most commonly used diagnostic test for SIBO as it is non-invasive and well tolerated. SIBO, as measured by GHBT, has been documented in 11% of Bangladeshi 18 week old children and the prevalence increases to 30–40% by 2 years of age [7,14]. Recent work in a Bangladeshi cohort showed that total bacterial load, as determined by 16s rRNA sequencing, in duodenal aspirate was associated with enteropathy in children [8]. Findings from Madagascar and the Central African Republic have shown an association between increased amounts of culturable total bacteria in the duodenum and stunted growth [15,16]. Additionally, key taxa of stunting and enteropathy were identified by both studies [15,16]. SIBO, as diagnosed by GHBT has also been associated with fecal markers of inflammation [14]. The GHBT has also been associated with linear growth stunting [14,17]. All of these studies have been cross-sectional. However, one longitudinal study in Bangladesh demonstrated SIBO in the first two years of life predicted future stunting and language delays [7].

Hydrogen breath testing measures excess hydrogen produced by the upper intestinal flora in response to an oral glucose load. This dysregulated hydrogen economy of the gut is not

present in all children with EED and the exact nature of this dysbiosis remains uncertain. Further, it is unclear if the dysbiosis detected by the GHBT is driven by lumenal shifts in bacteria or by shifts in those bacteria adherent to the intestinal wall. These populations are distinct and interact with the host differently [18].

All previous studies demonstrating a link between SIBO and EED have done so by demonstrating elevated biomarkers of inflammation in children with SIBO as compared to those without. However, no description of differences in histology between impoverished children with SIBO and those without has been published. Further, the only descriptions of the small intestinal microbiota associated with SIBO have involved study of duodenal aspirates with no description of the mucosa-adherent microbiota. They have also not used the GHBT for SIBO diagnosis which has been shown to predict future stunting and is associated with enteric inflammation [7–9]. Therefore, we conducted this study to test our hypotheses that the GHBT would be associated with EED as documented by histology and that the mucosa-adherent duodenal microbiome would differ significantly between impoverished SIBO positive and negative children.

Methods

Ethics statement

This project was approved by the Research Review Committee and the Ethics Review Committee at the International Centre for Diarrhoeal Disease Research, Bangladesh and the Institutional Review Board at the University of Virginia.

Study design and settings

This analysis was nested within the Bangladesh Environmental Enteric Dysfunction (BEED) study. BEED was a community-based interventional trial conducted in the slum setting of Mirpur in Dhaka, Bangladesh. This area is densely populated with >38,000 people living per square kilometer [19]. We enrolled children aged 12 to 18 months with a length-for-age Z score (LAZ) ≤-1 SD. This cutoff was chosen for the larger BEED study as indicative of children with chronic malnutrition. Participants were excluded if they were exclusively breastfed, had severe acute malnutrition, severe anemia, tuberculosis, any congenital anomaly or deformity, any severe or chronic disease, an ongoing episode of diarrhea, or a history of persistent diarrhea in the month preceding enrollment. All children received a directly-observed nutritional therapy of an egg and 150 ml milk for 90 days. Subsequently, a subset underwent esophagogastroduodenoscopy (EGD) if their LAZ failed to improve by the end of the 90 day intervention. Enrolled children also had GHBT for detection of SIBO within 7 days of EGD. The detailed procedure of GHBT and the methodology of the BEED study have been published [20,21]. EGD was performed on 120 children, of which 118 also had the GHBT. We have included those 118 children in this analysis. However, due to a cold shipment failure effecting 65 samples, we could not analyze the microbiome data from all 118 children. Data from 53 children were included in the microbiome analysis (S1 Fig).

The BEED study was approved by the Institutional Review Board of the International Centre for Diarrhoeal Disease Research, Bangladesh. All methods were carried out in accordance with relevant guidelines and regulations. Written informed consent was obtained from the parents or legal guardians at enrollment as well as prior to EGD. Biopsy samples for 16S rRNA analysis were sent to the UVa under a materials transfer agreement with the icddr,b, Dhaka, Bangladesh.

Data collection

Socioeconomic and household information was obtained from the parents or caregivers of the children in the Bangladeshi cohort at enrollment. Anthropometry data was collected by trained field staff using standard procedures based on the manuals of World Health Organization (WHO) and Centers for Disease Control and Prevention (CDC) guidelines. LAZ was calculated using WHO anthropometry software. EGD was performed by gastroenterologists with expertise in the clinical care of childhood digestive disorders. Two biopsy tissues were obtained from each child, one from the second part of the duodenum (D1) and another from the duodenal bulb (D2). Biopsy tissues were oriented immediately after collection under a microscope. Biopsies were placed in AllProtect (Qiagen, Inc. Hilden, Germany) immediately after collection, and stored at -80 C until bacterial DNA extraction.

For histological analysis, biopsies were formalin-fixed, paraffin-embedded, and then sectioned. A consortium of pathologists, independent from study investigators, was assembled for this study. This consortium developed an index of histopathological findings of EED and scored tissue slides [22]. This novel histological index consists of 11 components: Acute inflammation, chronic inflammation, eosinophil infiltration, intra-epithelial lymphocytes, villous architecture, intra-mucosal Brunner glands (which secrete mucin and epidermal growth factor), foveolar cell (secrete mucin) metaplasia, goblet cell (secretes mucus) density, Paneth cell (secrete antimicrobial peptides and proteins) density, enterocyte injury, and epithelial detachment (S1 Table) [23–26].

SIBO testing

SIBO was assessed by GHBT in the 7 days prior to EGD. Children who were acutely ill or had received antibiotics in the 14 days preceding a scheduled GHBT were rescheduled until after the 14-day period or illness had resolved, whichever was longer. Children were fasted for 2 hours prior to GHBT. We collected a baseline breath sample and then administered a glucose solution of 100g glucose in 500 ml sterile water administered at 5 ml/kg body weight over 10 minutes. Breath was then collected every 20 minutes for 3 hours. Samples were collected using the Quintron (Milwaukee, WI, USA) child breath collection bag and one-way flutter valve which was connected to an appropriately sized pediatric anesthesia mask. Breath samples were immediately analyzed using a Quintron BreathTracker SC (Milwaukee, WI, USA) gas chromatograph. Samples with lower than expected CO2, per the manufacturer's protocol, were considered contaminated with room air, discarded, and immediately recollected. Children were allowed only water during the fasting and testing periods. The GHBT results were dichotomized to positive or negative with a child was labeled as SIBO positive if they had a single post-glucose hydrogen reading ≥12 ppm over their baseline value. This cutoff was chosen prior to the publication of the American College of Gastroenterology Clinical Guideline for Small Intestinal Bacterial Overgrowth based on data demonstrating a decreased sensitivity when higher cutoffs are used [27–30]. However, given recent analysis showing the trapezoidal area under the hydrogen curve (SIBO AUC) was a better predictor of linear growth in children with EED, SIBO AUC was also utilized to study relationships between the GHBT, duodenal histology, and the mucosa-associated microbiota [7].

Statistical analysis

Statistical analyses were done using R version 3.5.3 (https://www.rproject.org, Foundation for Statistical Computing, Vienna, Austria) software. The baseline characteristics of the children were described using number with proportion for categorical variables. Mean ± standard deviation (SD) were used for normally distributed continuous variables. The asymmetric

numeric variables are reported with median and inter-quartile range (IQR). The components of histological index were compared between SIBO-positive and SIBO-negative children using Wilcoxon–Mann–Whitney tests as a dichotomous cut-off is the standard interpretation of the glucose hydrogen breath test [29,30]. As an exploratory endeavour, based on our previous findings using SIBO AUC, we then determined the correlation between SIBO AUC and components of the histological index using Spearman's rank correlation test [7].

The V4 region of the 16S gene was amplified and sequenced using the primer sequences (S2 Table) and methodology published by Kozich, et al. and was done in triplicate [31]. The library was processed using the DADA2 (v. 1.16.0) pipeline and taxonomy was assigned using Silva v.138. Reads were truncated at 190 base pairs for forward reads and 170 base pairs for reverse reads, and a pair-end library was used with expected size of 250 base pairs. Rare taxa that did not have at least 3 copies present in at least 5% of the samples were removed. One sample which only has 3 reads was removed from analysis.

Samples were rarefied using 'rarefy_even_depth' function in the R package 'phyloseq'. Shannon index was used to calculate alpha diversity (the intra-sample diversity). The significance of differences in alpha diversity across groups was tested using the Wilcoxon rank sum test implemented in the R function wilcox.test. A Brays-Curtis dissimilarity Principle Coordinates Analysis (PCoA) on taxa relative abundance was used for visualization of beta diversity (the intra-group diversity).

We assessed for taxa differences in children who were SIBO positive (i.e. >12 ppm increase in exhaled H2 over baseline reading) and negative. We then tested for differences across the 11 histological aspects scored. The significance between taxa relative abundance Bray-Curtis distances and SIBO or histology was evaluated using univariate PERMANOVA tests on Bray-Curtis distance using R function 'adonis()' in package 'vegan' [32]. As there was insufficient power to test for differences in SIBO positivity and negativity using PERMANOVA, we divided the children based on the SIBO AUC into the top and bottom 50th percentiles. These groups, as well has histological features that had a PERMANOVA p value <0.1 were analyzed to identify differentially abundant taxa. Univariate taxa differential abundance in two groups of samples was tested using a negative binomial model for the overdispersion and Poisson process intrinsic to microbiome data, as implemented in DESeq2 package in R, utilizing the Benjamini and Hochberg method for multiple testing correction [33]. Initial models were then repeated, correcting for breastfeeding status (some vs. none).

Results

One hundred eighteen Bangladeshi children underwent EGD and GHBT. No adverse events due to EGD occurred. Of them, 53 children had 16s rRNA data available. One child had only 100 reads and was removed from further analysis. Twenty-two (41.5%) children were male, and their mean (±SD) age was 18.7 (±2.1) months. Overall, 15 (12.7%) of the children tested positive for SIBO. Of the 53 children with 16s rRNA data available, 8 (15.1%) tested SIBO positive. The socio-demographic information of the children is presented in Table 1.

Histological differences by GHBT results

No significant difference was observed for the scores of the 11 histological elements between SIBO-positive and SIBO-negative children when GHBT output was dichotomized based on the 12 ppm cutoff. SIBO AUC was positively correlated with the score of enterocyte injury in the second part of the duodenum ($R = 0.21$, $p = 0.02$) (Fig 1). The median enterocyte injury score in the second part of the duodenum was 0.30 with an interquartile range of 0.00–0.50. Linear regression was used to determine the linear trend in the association between AUC

Table 1. Baseline Characteristics of the Children with and without 16s rRNA profiling.

	Children with 16s rRNA Data (n = 53)	Children without 16s rRNA Data (n = 65)
Age (days)*	545 [512.0, 615.0]	566.0 [512.0, 617.0]
Male#	22 (41.5%)	26 (40.0%)
Any breastfeeding*$	46 (86.8%)	51 (78.5%)
LAZ*	-2.24 [-2.86, -1.73]	-2.0 [-2.7, -1.4]
WAZ*	-2.22 [-2.57, -1.62]	-1.47 [-1.87, -0.80]
WLZ*	-1.32 [-1.90, -0.87]	-0.57 [-1.14, -0.15]
SIBO Positive#	8 (15.1%)	7 (10.8%)
SIBO AUC (ppm hydrogen)*	1240.0 [840.0, 1970.0]	1100.0 [690.0, 1770.0]
WAMI Score*	0.6 [0.5, 0.6]	0.6 [0.5, 0.7]
Monthly family income <200 USD#	42 (79.2%)	43 (66.2%)
Improved source of drinking water#	53 (100%)	65 (100%)
Improved sanitation#	40 (75.5%)	44 (67.7%)
Household crowding (>4 people sleep in a room)#	10 (18.9%)	11 (16.9%)
Villous atrophy#	16 (30.2%)	54 (83.1%)
Crypt hyperplasia#	15 (28.3%)	42 (64.6%)
Inflammatory infiltrates in lamina propria#	48 (90.6%)	64 (98.5%)

*Median (Q1, Q3)

#Count (%)

Abbreviations: LAZ, length-for-age Z score; WAZ, weight-for-age Z score; WLZ, weight-for-length Z score; SIBO, small intestine bacterial overgrowth; AUC, area under the curve; ppm, parts per million; WAMI, water & sanitation, assets, maternal education and household income.

$N.B. No children were exclusively breastfed.

https://doi.org/10.1371/journal.pntd.0012023.t001

and enterocyte injury score (Fig 1C). Based on this analysis, for every 1 ppm increase in SIBO AUC, the enterocyte injury score increased by 0.0003 in the second part of the duodenum. The average SIBO AUC in the cohort was 1467 ppm (range 160 to 5960) meaning the average child had an increase of 0.44 points in enterocyte injury score due to SIBO while the most severe case had an increase of 1.7 points. None of the other components had a statistically significant correlation with SIBO AUC (S2 Fig).

16S rRNA analysis

A median of 15,792 raw reads per sample (range 3–184,266) was generated from the biopsy samples. After removing rare taxa and the sample with 3 total reads, a median of 15,812 reads remained (range 2,151–184,266). There were 117 unique amplicon sequence variants (ASVs) identified. ASVs in children in the top and bottom 50th percentile for SIBO AUC are shown in Fig 2 (S3 Table).

Microbiome of SIBO in Bangladeshi children

There was no difference in alpha diversity of the microbiota as measured by Shannon Diversity index between SIBO positive and negative children (median 2.55 vs. 2.44, p = 0.67). A PCoA plot did not demonstrate separation between SIBO negative and positive children (Fig 3A). PERMANOVA p value was 0.74 comparing the top and bottom 50th percentiles for SIBO AUC. Differential abundance analysis did not select any taxa predictive of SIBO

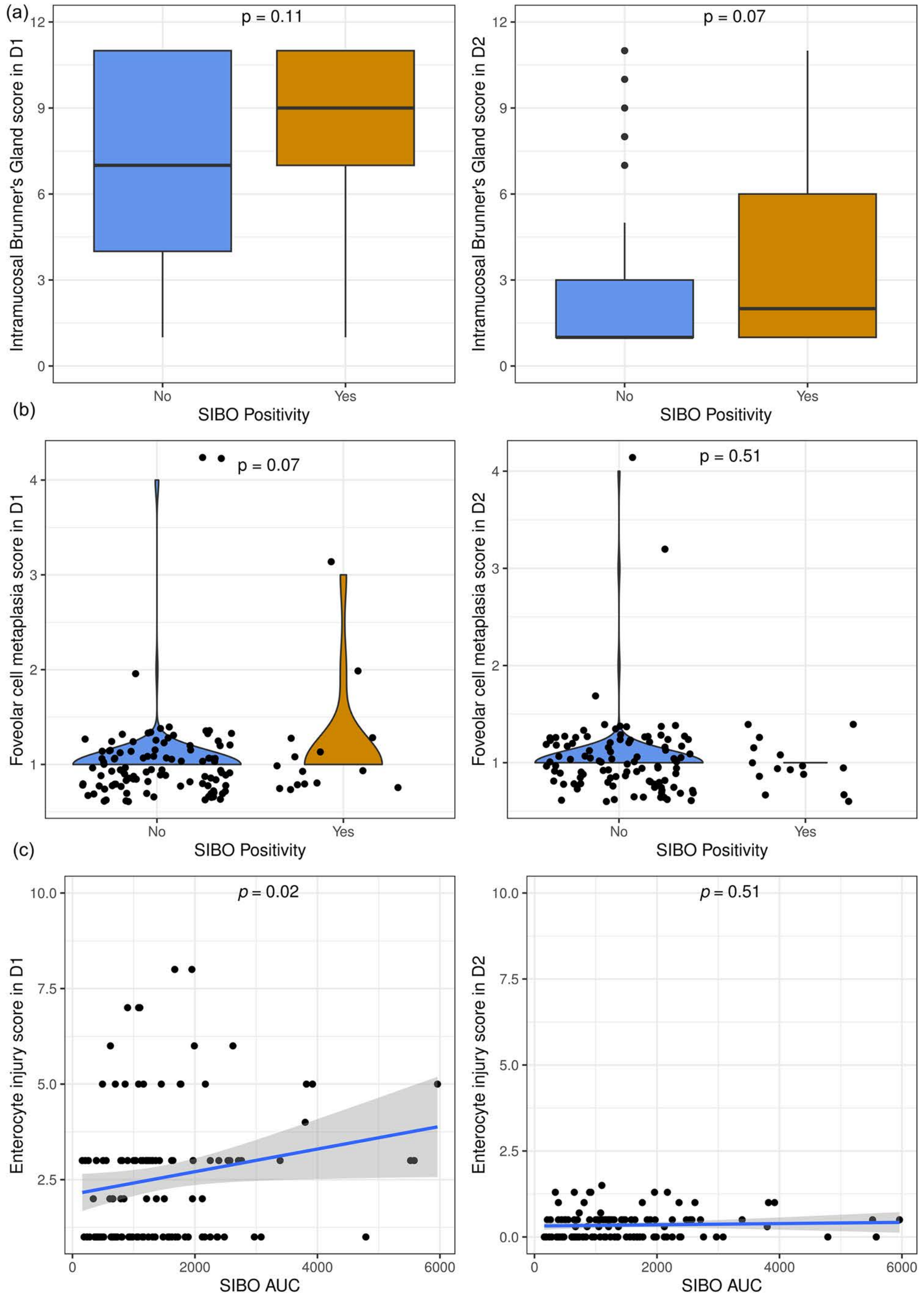

Fig 1. Histologic comparison of SIBO positive and negative children. Histologic assessment of duodenal biopsies from children with and without SIBO demonstrated increases in intramucosal Bruner's Glands (p = 0.07) (panel A) and foveolar cell metaplasia (p = 0.07) (panel B) in the second and first parts of the duodenum, respectively, in children who were SIBO positive. Enterocyte injury directly correlated with SIBO AUC in the first part of the duodenum (p = 0.02) (panel C).

https://doi.org/10.1371/journal.pntd.0012023.g001

positivity. Children in the upper 50th percentile for SIBO AUC had a high abundance of genus *Campylobacter* (Log 2-fold change of 4.43; adjusted p = 1.9 x 10^{-6}) compared to those in the lower 50th percentile (Fig 4A). PERMANOVA analysis determined only crypt hyperplasia

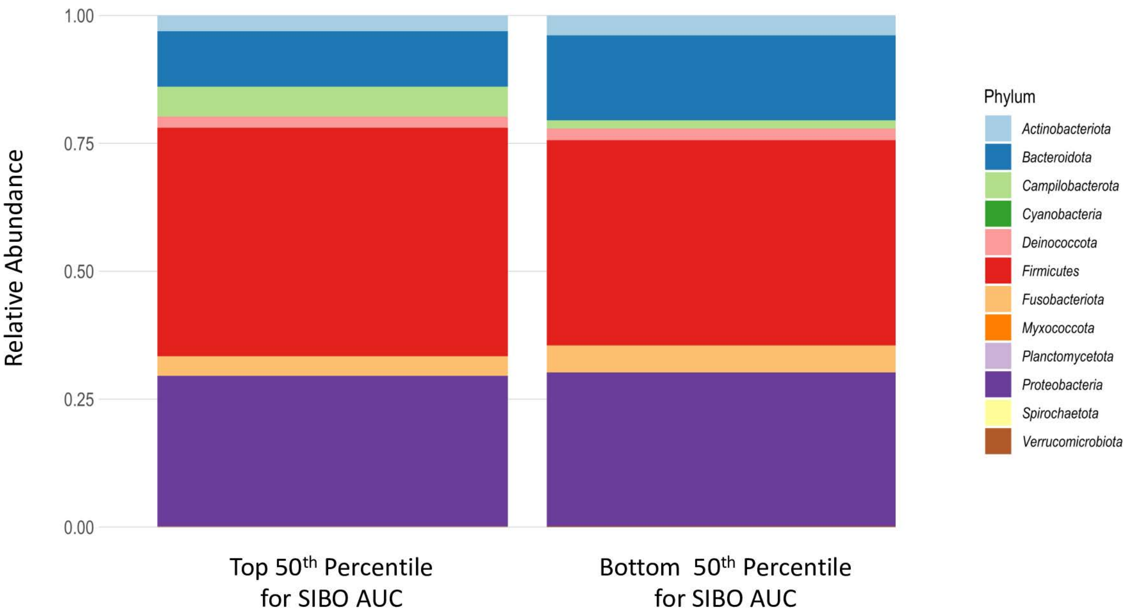

Fig 2. Phylum comparison between SIBO status. Phylum level determinations are depicted for both the top and both 50th percentile for SIBO AUC. DESeq2 only selected the genus Campylobacter as discriminating these two groups.

https://doi.org/10.1371/journal.pntd.0012023.g002

(p = 0.05), villous atrophy (p = 0.08), and foveolar cell metaplasia (p = 0.03) to be associated with features of the adherent microbiome. There was no difference in alpha diversity between children with and without villous atrophy (median Shannon Diversity Index 2.58 vs 2.38, p = 0.12) or with and without crypt hyperplasia (median 2.55 vs 2.41, p = 0.21) (Fig 3B and 3C). Foveolar cell metaplasia was not analyzed as there were only 3 subjects with values other than zero despite significance on PERMANOVA. *Campylobacter* was the only genera associated with histological findings. Children with no crypt hyperplasia had higher *Campylobacter* abundance compared to those with crypt hyperplasia (4.35 Log 2-fold change; adjusted p value 4.6x 10^{-4}). Children with no villous atrophy had a higher *Campylobacter* abundance compared to those with villous atrophy (4.45 Log 2-fold change; adjusted p value 1.5x 10^{-4}) (Fig 4B and 4C).

In models corrected for breastfeeding status, no taxa were significantly associated with SIBO status, SIBO AUC (top vs bottom 50th percentile), villous atrophy, or crypt hyperplasia.

Discussion

This work is the first to investigate duodenal histology and the epithelial adherent microbiota of the duodenum in relation to glucose hydrogen breath testing in a low-income setting. The main finding was that increased SIBO AUC was associated with enterocyte injury. A growing body of literature has described high rates of GHBT positivity in children from low-incomes countries [7,14,34–36]. Recent work shown GHBT positivity to be a risk factor for linear growth stunting and language delay [7]. SIBO, as diagnosed by GHBT, has been associated with several markers of intestinal inflammation including fecal Reg1B (an anti-apoptotic, pro-proliferative protein secreted by damaged epithelial cells) and fecal myeloperoxidase (a marker of neutrophilic infiltrate in the gut) [14]. SIBO, as determined by 16s rDNA read count in duodenal aspirate, has also been associated with systemic inflammatory cytokines [8]. SIBO, defined by culture of duodenal aspirates, has been associated with increased fecal calprotectin and alpha-1-antitrypsin with the culturable SIBO microbiota leading to decreased lipid absorption when transplanted to murine intestinal cells [9]. This work furthers these

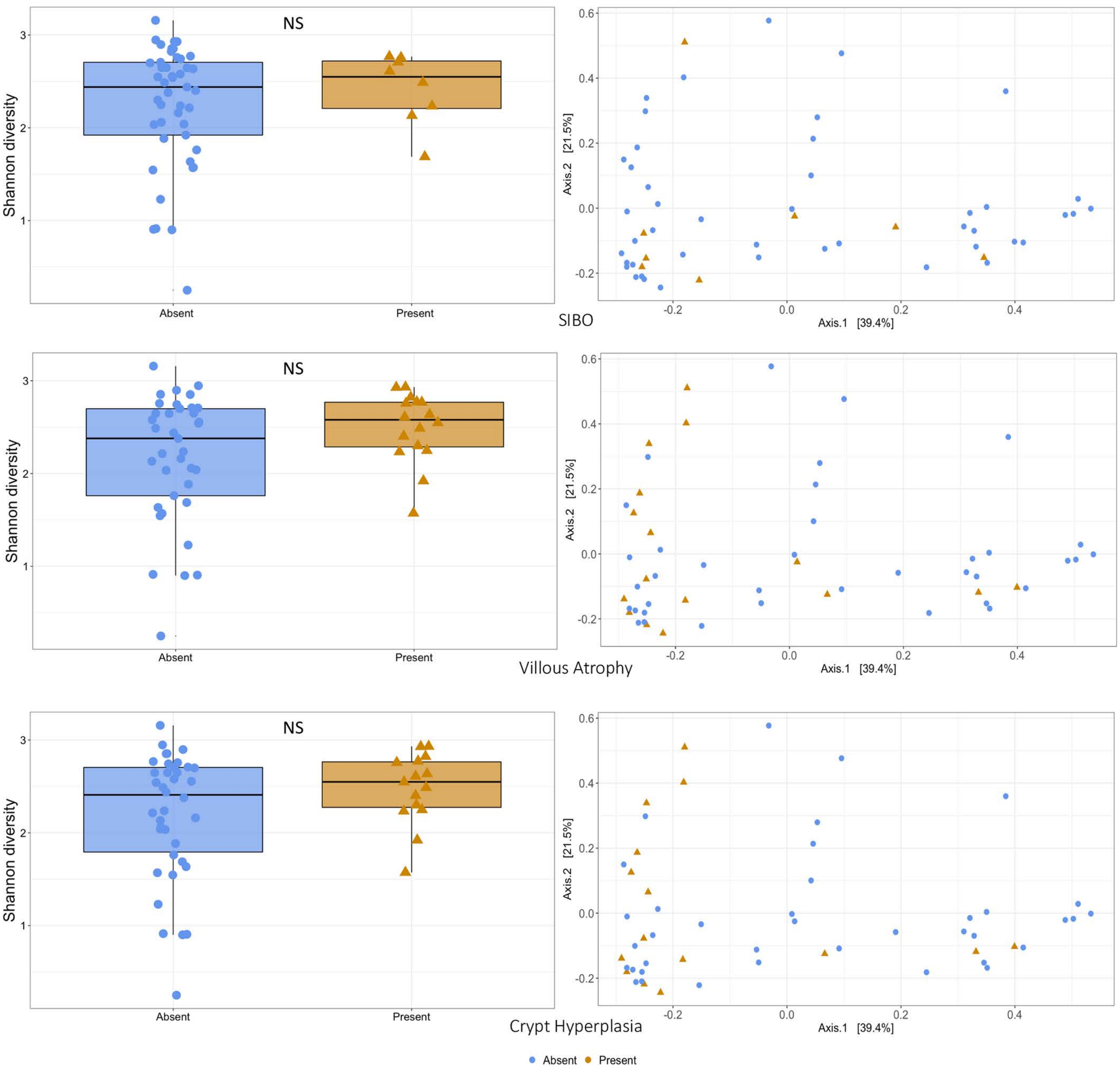

Fig 3. Alpha and Beta diversity analysis for children with and without SIBO, villous atrophy, and crypt hyperplasia. Children with and without SIBO, villous atrophy, and crypt hyperplasia were assessed for differences in the Shannon alpha diversity index using Wilcoxon–Mann–Whitney tests. No significant differences (NS) were noted. PCoA plots were constructed for each variable without notable separation of positive and negative children.

https://doi.org/10.1371/journal.pntd.0012023.g003

findings by showing a direct association of GHBT positivity and epithelial damage while showing GHBT positivity is not associated with increases in the classic markers of EED histology (i.e. crypt hyperplasia, villous blunting, and inflammatory infiltrate into the lamina propria). While the observed statistically significant effect size seems small, correlation to clinically significant outcomes such as growth or neurodevelopment is unknown.

Recent work describing the histology of EED has shown eight of the 11 parameters utilized in our analysis to have sufficient variance to be useful in the assessment of EED in children

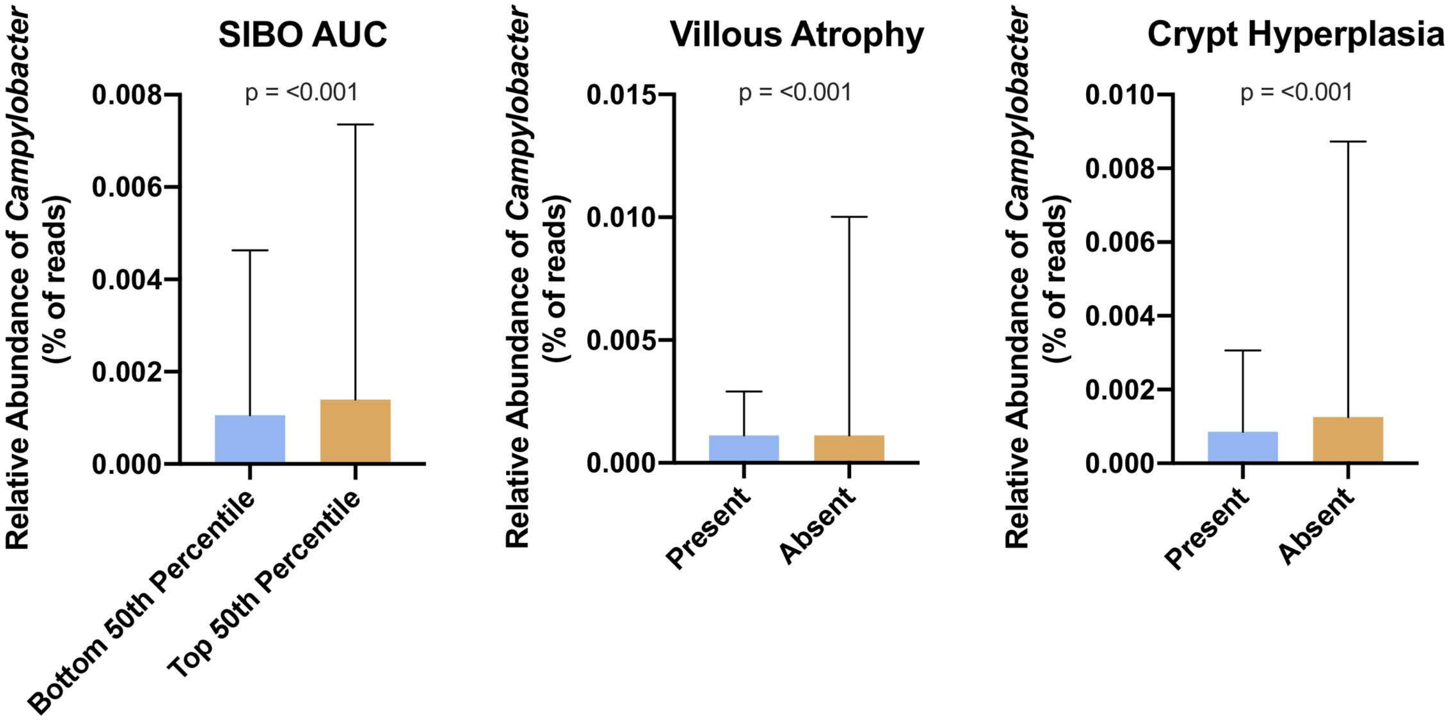

Fig 4. Association of *Campylobacter* spp. with SIBO and duodenal histology. *Campylobacter* reads were higher in children with a SIBO AUC in the top 50th percentile and those without villous atrophy or crypt hyperplasia. Data are displayed as median and upper limits of the interquartile range (whiskers).

https://doi.org/10.1371/journal.pntd.0012023.g004

[22]. While villous atrophy, crypt hyperplasia, and lymphocytic infiltrate have been the traditional histologic hallmarks of EED, recent evidence suggests depletion of Paneth and goblet cells is also a predominating feature [3]. Transcriptomic analysis has shown dysregulated lipid metabolism to be common in children with EED [37]. Further, distinct bile acid signatures have been identified in children with EED [38]. This cumulative evidence strongly implies a small intestinal dysbiosis is pathogenic in children with EED [3].

Several studies have used duodenal sampling to diagnose SIBO and have described microbial populations in the luminal aspirate [8,16]. These studies have also shown an association between SIBO and stunted growth. This work is the first to investigate the epithelial adherent microbiota in this setting. *Campylobacter* was increased in patients with higher SIBO AUC although this associated was not shown in a model corrected for breastfeeding status. This likely reflects an interaction between breastfeeding and the microbiota which decreased our power to detect significant associations, especially given our low sample size. A potential link between *Campylobacter* and SIBO has been described whereby *Campylobacter* can induce anti- Cytolethal Distending Toxin (CDT) antibodies which cross react with vinculin in the enteric nervous system leading to irritable bowel symptoms. In a rat model, anti-CDT titers were associated with the development of SIBO [28]. This suggests that epithelial adherent *Campylobacter* can precipitate SIBO by an immune mediated mechanism [39,40]. The cumulative burden of *Campylobacter* infection in children from low-income countries has been directly associated with chronic malnutrition and stunting [41,42]. Contrary to what we would have expected based on this literature, we detected less *Campylobacter* in patients with villous atrophy or crypt hyperplasia. *Campylobacter's* pathogenesis depends on multiple virulence factors responsible for adherence including CadF which binds epithelial fibronectin and is essential for invasion [43]. The helical shape of *Campylobacter* specifically allows the organism

to target crypts for adhesion and colonization [44]. It may be that an unhealthy epithelium with malformed crypts represents a barrier to *Campylobacter* adhesion or that epithelial turn-over is a defense mechanism against *Campylobacter*. Thus, while *Campylobacter* infection may be an inflammatory insult creating epithelial cell damage, the organism may struggle to adhere to a previously damaged small intestine. *Campylobacter* typically colonizes the distal small intestine and large intestine and thus our findings may be specific to the proximal small bowel thus limiting the conclusions we can draw on *Campylobacter* pathogenesis [45,46].

Our study had several notable strengths. First, SIBO was assessed by GHBT, a biomarker that has been directly associated with poor linear growth. Our pathological assessment was robust, using a validated scoring system by multiple pathologists. Finally, the collection of specimens was protocolized to preserve nucleic acid. There are also several important limitations that should be considered when interpreting findings from this analysis. Our sample size was relatively small which may limit our ability to detect smaller effect sizes, especially when covariates such as breastfeeding were included in our models. Our measure of breast-feeding (some vs none) was crude where more robust breastfeeding data would likely offer valuable insights into the relationship breastfeeding may play between the enteric microbiota and our outcomes of interest. Biopsy samples also had lower read counts compounding the effects of low sample size. Biopsy sampling involved only two samples from each patient and it is unknown if EED has patchy disease similar to celiac disease or inflammatory bowel disease which could have led to missed pathology. Also, it should be emphasized that our work examined only the mucosa-adherent microbiome obtained from biopsy samples. The luminal microbiome may play a significant role in SIBO pathogenesis and breath hydrogen test positivity which involve taxa not detected in our analysis. Finally, we excluded children with severe acute malnutrition which likely represents the most extreme end of the EED spectrum and thus there is an element of selection bias to this study.

Conclusions

The findings of this work suggest that SIBO may be associated with epithelial cell damage and *Campylobacter* colonization of the epithelial layer of the small intestine. Further work is needed to better understand the relationship between SIBO, EED, and *Campylobacter* infection as SIBO may be a modifiable risk factor leading to malnutrition and neurodevelopmental delay.

Supporting information

S1 Fig. Flowchart of study enrollment. 118 children enrolled in the original Bangladesh Environmental Enteric Diseases (BEED) study had glucose-hydrogen breath testing for SIBO with 53 of those having 16s rRNA sequencing conducted on duodenal biopsy samples.
(TIF)

S2 Fig. Comparison of histologic features by SIBO AUC. Enterocyte injury (Fig 1) was the only significant feature. No other features demonstrated a significant association.
(TIF)

S1 Table. The histologic scoring index used by the consortium of pathologists utilized 11 parameters to score duodenal biopsy samples.
(DOCX)

S2 Table. The primers used for V4 amplification, first published by Kozich et al. [31].
(DOCX)

S3 Table. The average relative abundances of all detected taxa are displayed for all children for whom 16s rRNA data is available (Column B). Also displayed are the average relative abundances for children in the top 50th percentile (Column C) and the bottom 50th percentile for SIBO AUC (Column D). Rare taxa that did not have at least 3 copies present in at least 5% of the samples were removed.
(XLSX)

Acknowledgments

The authors express thanks to the staff and participants of the BEED study as well as the field and laboratory staff at icddr,b for their valuable contributions. We are grateful to the governments of Bangladesh, Canada, Sweden, and the UK for providing unrestricted support to the icddr,b.

Author contributions

Conceptualization: Shah Mohammad Fahim, Jeffrey R. Donowitz, Subhasish Das, Mustafa Mahfuz, William A. Petri, Chelsea Marie, Tahmeed Ahmed.

Data curation: Ning-Jiun Jan, Subhasish Das, Mustafa Mahfuz, S. M. Abdul Gaffar, Chelsea Marie.

Formal analysis: Shah Mohammad Fahim, Jeffrey R. Donowitz, Ekaterina Smirnova, Ning-Jiun Jan.

Funding acquisition: Jeffrey R. Donowitz, Subhasish Das, Mustafa Mahfuz, William A. Petri, Chelsea Marie, Tahmeed Ahmed.

Investigation: Shah Mohammad Fahim, Jeffrey R. Donowitz, Ning-Jiun Jan, Subhasish Das, Mustafa Mahfuz, S. M. Abdul Gaffar, William A. Petri, Chelsea Marie, Tahmeed Ahmed.

Methodology: Shah Mohammad Fahim, Jeffrey R. Donowitz, Ekaterina Smirnova, Ning-Jiun Jan, Subhasish Das, Mustafa Mahfuz, William A. Petri, Chelsea Marie, Tahmeed Ahmed.

Project administration: Shah Mohammad Fahim, Jeffrey R. Donowitz, Subhasish Das, Mustafa Mahfuz, Chelsea Marie, Tahmeed Ahmed.

Supervision: Jeffrey R. Donowitz, Subhasish Das, Mustafa Mahfuz, William A. Petri, Chelsea Marie, Tahmeed Ahmed.

Visualization: Ekaterina Smirnova.

Writing – original draft: Shah Mohammad Fahim, Jeffrey R. Donowitz, Ekaterina Smirnova.

Writing – review & editing: Shah Mohammad Fahim, Jeffrey R. Donowitz, Ekaterina Smirnova, William A. Petri, Chelsea Marie, Tahmeed Ahmed.

References

1. Harper KM, Mutasa M, Prendergast AJ, Humphrey J, Manges AR. Environmental enteric dysfunction pathways and child stunting: A systematic review. PLoS Negl Trop Dis. 2018;12: e0006205. https://doi.org/10.1371/journal.pntd.0006205 PMID: 29351288
2. Fahim SM, Das S, Gazi MdA, Mahfuz M, Ahmed T. Association of intestinal pathogens with faecal markers of environmental enteric dysfunction among slum-dwelling children in the first 2 years of life in Bangladesh. Tropical Medicine & International Health. 2018;23: 1242–1250. https://doi.org/10.1111/tmi.13141 PMID: 30133067
3. Hodges P, Tembo M, Kelly P. Intestinal Biopsies for the Evaluation of Environmental Enteropathy and Environmental Enteric Dysfunction. J Infect Dis. 2021;224: S856–S863. https://doi.org/10.1093/infdis/jiab372 PMID: 34273148

4. Watanabe K, Petri WA. Environmental Enteropathy: Elusive but Significant Subclinical Abnormalities in Developing Countries. EBioMedicine. 2016;10: 25–32. https://doi.org/10.1016/j.ebiom.2016.07.030 PMID: 27495791
5. Owino V, Ahmed T, Freemark M, Kelly P, Loy A, Manary M, et al. Environmental Enteric Dysfunction and Growth Failure/Stunting in Global Child Health. Pediatrics. 2016;138: e20160641–e20160641. https://doi.org/10.1542/peds.2016-0641 PMID: 27940670
6. Guerrant RL, DeBoer MD, Moore SR, Scharf RJ, Lima AAM. The impoverished gut—a triple burden of diarrhoea, stunting and chronic disease. Nature Reviews Gastroenterology and Hepatology. 2013;10: 220–229. https://doi.org/10.1038/nrgastro.2012.239 PMID: 23229327
7. Donowitz JR, Pu Z, Lin Y, Alam M, Ferdous T, Shama T, et al. Small Intestine Bacterial Overgrowth in Bangladeshi Infants Is Associated With Growth Stunting in a Longitudinal Cohort. Am J Gastroenterol. 2021. https://doi.org/10.14309/ajg.0000000000001535 PMID: 34693912
8. Chen RY, Kung VL, Das S, Hossain MS, Hibberd MC, Guruge J, et al. Duodenal Microbiota in Stunted Undernourished Children with Enteropathy. N Engl J Med. 2020;383: 321–333. https://doi.org/10.1056/NEJMoa1916004 PMID: 32706533
9. Vonaesch P, Araújo JR, Gody J-C, Mbecko J-R, Sanke H, Andrianonimiadana L, et al. Stunted children display ectopic small intestinal colonization by oral bacteria, which cause lipid malabsorption in experimental models. Proc Natl Acad Sci U S A. 2022;119: e2209589119. https://doi.org/10.1073/pnas.2209589119 PMID: 36197997
10. Fahim SM, Das S, Gazi MA, Alam MA, Hasan MM, Hossain MS, et al. Helicobacter pylori infection is associated with fecal biomarkers of environmental enteric dysfunction but not with the nutritional status of children living in Bangladesh. PLoS Negl Trop Dis. 2020;14: e0008243. https://doi.org/10.1371/journal.pntd.0008243 PMID: 32324737
11. Fahim SM, Das S, Gazi MA, Alam MA, Mahfuz M, Ahmed T. Evidence of gut enteropathy and factors associated with undernutrition among slum-dwelling adults in Bangladesh. Am J Clin Nutr. 2020;111: 657–666. https://doi.org/10.1093/ajcn/nqz327 PMID: 31909785
12. Tickell KD, Atlas HE, Walson JL. Environmental enteric dysfunction: a review of potential mechanisms, consequences and management strategies. BMC Med. 2019;17: 181. https://doi.org/10.1186/s12916-019-1417-3 PMID: 31760941
13. Donowitz JR, Petri WA. Pediatric small intestine bacterial overgrowth in low-income countries. Trends in Molecular Medicine. 2015;21: 6–15. https://doi.org/10.1016/j.molmed.2014.11.001 PMID: 25486880
14. Donowitz JR, Haque R, Kirkpatrick BD, Alam M, Lu M, Kabir M, et al. Small Intestine Bacterial Overgrowth and Environmental Enteropathy in Bangladeshi Children. mBio. 2016;7: 10.1128/mBio.02102-15. https://doi.org/10.1128/mBio.02102-15 PMID: 26758185
15. Huus KE, Rodriguez-Pozo A, Kapel N, Nestoret A, Habib A, Dede M, et al. Immunoglobulin recognition of fecal bacteria in stunted and non-stunted children: findings from the Afribiota study. Microbiome. 2020;8: 113. https://doi.org/10.1186/s40168-020-00890-1 PMID: 32718353
16. Vonaesch P, Morien E, Andrianonimiadana L, Sanke H, Mbecko J-R, Huus KE, et al. Stunted childhood growth is associated with decompartmentalization of the gastrointestinal tract and overgrowth of oropharyngeal taxa. Proceedings of the National Academy of Sciences of the United States of America. 2018; 201806573–201806573. https://doi.org/10.1073/pnas.1806573115 PMID: 30126990
17. Mello CS, Rodrigues MS do C, Filho HB de A, Melli LCFL, Tahan S, Pignatari ACC, et al. Fecal microbiota analysis of children with small intestinal bacterial overgrowth among residents of an urban slum in Brazil. Jornal de pediatria. 2017. https://doi.org/10.1016/j.jped.2017.09.003 PMID: 29049893
18. Chen W, Liu F, Ling Z, Tong X, Xiang C. Human Intestinal Lumen and Mucosa-Associated Microbiota in Patients with Colorectal Cancer. PLOS ONE. 2012;7: e39743. https://doi.org/10.1371/journal.pone.0039743 PMID: 22761885
19. Das S, Rasul MG, Hossain MS, Khan A-R, Alam MA, Ahmed T, et al. Acute food insecurity and short-term coping strategies of urban and rural households of Bangladesh during the lockdown period of COVID-19 pandemic of 2020: report of a cross-sectional survey. BMJ Open. 2020;10: e043365. https://doi.org/10.1136/bmjopen-2020-043365 PMID: 33310813
20. Mahfuz M, Das S, Mazumder RN, Masudur Rahman M, Haque R, Bhuiyan MMR, et al. Bangladesh Environmental Enteric Dysfunction (BEED) study: protocol for a community-based intervention study to validate non-invasive biomarkers of environmental enteric dysfunction. BMJ Open. 2017;7: e017768. https://doi.org/10.1136/bmjopen-2017-017768 PMID: 28801442
21. Mahfuz M, Sarker SA, Ahmed T, Gaffar SMA, Donowitz JR. Impact of Small Intestine Bacterial Overgrowth on Response to a Nutritional Intervention in Bangladeshi Children from an Urban Community.

The American Journal of Tropical Medicine and Hygiene. 2018;100: tpmd180759–tpmd180759. https://doi.org/10.4269/ajtmh.18-0759 PMID: 30479249

22. Liu T-C, VanBuskirk K, Ali SA, Kelly MP, Holtz LR, Yilmaz OH, et al. A novel histological index for evaluation of environmental enteric dysfunction identifies geographic-specific features of enteropathy among children with suboptimal growth. PLOS Neglected Tropical Diseases. 2020;14: e0007975. https://doi.org/10.1371/journal.pntd.0007975 PMID: 31929525
23. Lueschow SR, McElroy SJ. The Paneth Cell: The Curator and Defender of the Immature Small Intestine. Frontiers in Immunology. 2020;11. Available: https://www.frontiersin.org/articles/10.3389/fimmu.2020.00587 PMID: 32308658
24. Krause WJ. Brunner's Glands: A Structural,Histochemical and Pathological Profile. Progress in Histochemistry and Cytochemistry. 2000;35: 255–367. https://doi.org/10.1016/S0079-6336(00)80006-6
25. Ootani A, Toda S, Fujimoto K, Sugihara H. Foveolar Differentiation of Mouse Gastric Mucosa in Vitro. Am J Pathol. 2003;162: 1905–1912. https://doi.org/10.1016/S0002-9440(10)64324-6 PMID: 12759247
26. Gustafsson JK, Johansson MEV. The role of goblet cells and mucus in intestinal homeostasis. Nat Rev Gastroenterol Hepatol. 2022;19: 785–803. https://doi.org/10.1038/s41575-022-00675-x PMID: 36097076
27. Donald IP, Kitchingmam G, Donald F, Kupfer RM. The diagnosis of small bowel bacterial overgrowth in elderly patients. J Am Geriatr Soc. 1992;40: 692–696. https://doi.org/10.1111/j.1532-5415.1992.tb01961.x PMID: 1607585
28. Stotzer PO, Kilander AF. Comparison of the 1-gram (14)C-D-xylose breath test and the 50-gram hydrogen glucose breath test for diagnosis of small intestinal bacterial overgrowth. Digestion. 2000;61: 165–171. https://doi.org/10.1159/000007753 PMID: 10773721
29. Gasbarrini A, Corazza GR, Gasbarrini G, Montalto M, Di Stefano M, Basilisco G, et al. Methodology and indications of H2-breath testing in gastrointestinal diseases: the Rome Consensus Conference. Alimentary Pharmacology & Therapeutics. 2009;29 Suppl 1: 1–49. https://doi.org/10.1111/j.1365-2036.2009.03951.x PMID: 19344474
30. Pimentel M, Saad RJ, Long MD, Rao SSC. ACG Clinical Guideline: Small Intestinal Bacterial Overgrowth. Am J Gastroenterol. 2020;115: 165–178. https://doi.org/10.14309/ajg.0000000000000501 PMID: 32023228
31. Kozich JJ, Westcott SL, Baxter NT, Highlander SK, Schloss PD. Development of a dual-index sequencing strategy and curation pipeline for analyzing amplicon sequence data on the MiSeq Illumina sequencing platform. Applied and Environmental Microbiology. 2013;79: 5112–5120. https://doi.org/10.1128/AEM.01043-13 PMID: 23793624
32. McArdle BH, Anderson MJ. Fitting Multivariate Models to Community Data: A Comment on Distance-Based Redundancy Analysis. Ecology. 2001;82: 290–297. https://doi.org/10.1890/0012-9658(2001)082[0290:FMMTCD]2.0.CO;2
33. Love MI, Huber W, Anders S. Moderated estimation of fold change and dispersion for RNA-seq data with DESeq2. Genome Biol. 2014;15: 550. https://doi.org/10.1186/s13059-014-0550-8 PMID: 25516281
34. Mello CS, Tahan S, Melli LCFL, do Carmo Rodrigues MS, de Mello RMP, Scaletsky ICA, et al. Methane production and small intestinal bacterial overgrowth in children living in a slum. World journal of gastroenterology: WJG. 2012;18: 5932–5932.
35. dos Reis JC, de Morais MB, Oliva CAG, Fagundes-Neto U. Breath hydrogen test in the diagnosis of environmental enteropathy in children living in an urban slum. Digestive diseases and sciences. 2007;52: 1253–1258. https://doi.org/10.1007/s10620-006-9288-9 PMID: 17372830
36. Pereira SP, Bolin TD, Duncombe VM, Linklater JM. A pattern of breath hydrogen excretion suggesting small bowel bacterial overgrowth in Burmese village children. Journal of pediatric gastroenterology and nutrition. 1991;13: 32–38. https://doi.org/10.1097/00005176-199107000-00006 PMID: 1833523
37. Haberman Y, Iqbal NT, Ghandikota S, Mallawaarachchi I, Braun Tzipi, Dexheimer PJ, et al. Mucosal Genomics Implicate Lymphocyte Activation and Lipid Metabolism in Refractory Environmental Enteric Dysfunction. Gastroenterology. 2021;160: 2055–2071.e0. https://doi.org/10.1053/j.gastro.2021.01.221 PMID: 33524399
38. Zhao X, Setchell KDR, Huang R, Mallawaarachchi I, Ehsan L, Dobrzykowski Iii E, et al. Bile Acid Profiling Reveals Distinct Signatures in Undernourished Children with Environmental Enteric Dysfunction. J Nutr. 2021;151: 3689–3700. https://doi.org/10.1093/jn/nxab321 PMID: 34718665
39. Pimentel M, Chatterjee S, Chang C, Low K, Song Y, Liu C, et al. A New Rat Model Links Two Contemporary Theories in Irritable Bowel Syndrome. Dig Dis Sci. 2008;53: 982–989. https://doi.org/10.1007/s10620-007-9977-z PMID: 17934822

40. Rezaie A, Park SC, Morales W, Marsh E, Lembo A, Kim JH, et al. Assessment of Anti-vinculin and Anti-cytolethal Distending Toxin B Antibodies in Subtypes of Irritable Bowel Syndrome. Dig Dis Sci. 2017;62: 1480–1485. https://doi.org/10.1007/s10620-017-4585-z PMID: 28451914

41. MAL-ED Network Investigators. Relationship between growth and illness, enteropathogens and dietary intakes in the first 2 years of life: findings from the MAL-ED birth cohort study. BMJ Glob Health. 2017;2: e000370. https://doi.org/10.1136/bmjgh-2017-000370 PMID: 29333282

42. Rogawski ET, Liu J, Platts-Mills JA, Kabir F, Lertsethtakarn P, Siguas M, et al. Use of quantitative molecular diagnostic methods to investigate the effect of enteropathogen infections on linear growth in children in low-resource settings: longitudinal analysis of results from the MAL-ED cohort study. Lancet Glob Health. 2018;6: e1319–e1328. https://doi.org/10.1016/S2214-109X(18)30351-6 PMID: 30287125

43. Bolton DJ. Campylobacter virulence and survival factors. Food Microbiol. 2015;48: 99–108. https://doi.org/10.1016/j.fm.2014.11.017 PMID: 25790997

44. Stahl M, Frirdich E, Vermeulen J, Badayeva Y, Li X, Vallance BA, et al. The Helical Shape of Campylobacter jejuni Promotes In Vivo Pathogenesis by Aiding Transit through Intestinal Mucus and Colonization of Crypts. Infect Immun. 2016;84: 3399–3407. https://doi.org/10.1128/IAI.00751-16 PMID: 27647867

45. Chang C, Miller JF. Campylobacter jejuni Colonization of Mice with Limited Enteric Flora. Infect Immun. 2006;74: 5261–5271. https://doi.org/10.1128/IAI.01094-05 PMID: 16926420

46. Colgan T, Lambert JR, Newman A, Luk SC. Campylobacter jejuni enterocolitis. A clinicopathologic study. Arch Pathol Lab Med. 1980;104: 571–574. PMID: 6893534
